# Identification of condition-specific regulatory mechanisms in normal and cancerous human lung tissue

**DOI:** 10.1186/s12864-022-08591-9

**Published:** 2022-05-06

**Authors:** Yuqing Hang, Josh Burns, Benjamin T. Shealy, Rini Pauly, Stephen P. Ficklin, Frank A. Feltus

**Affiliations:** 1 Department of Genetics & Biochemistry, Clemson University, Clemson, 29634 USA; 2 Department of Horticulture, Washington State University, Pullman, 99164 USA; 3 Department of Electrical and Computer Engineering, Clemson University, Clemson, 29634 USA; 4 Biomedical Data Science and Informatics Program, Clemson University, Clemson, 29634 USA; 5 Center for Human Genetics, Clemson University, Clemson, 29634 USA; 6 Biosystems Research Complex, 302C, 105 Collings St, Clemson, SC 29634 USA

**Keywords:** Lung cancer, Gene co-expression network, Gene regulatory network, Regulation, Biomarker system

## Abstract

**Background:**

Lung cancer is the leading cause of cancer death in both men and women. The most common lung cancer subtype is non-small cell lung carcinoma (NSCLC) comprising about 85% of all cases. NSCLC can be further divided into three subtypes: adenocarcinoma (LUAD), squamous cell carcinoma (LUSC), and large cell lung carcinoma. Specific genetic mutations and epigenetic aberrations play an important role in the developmental transition to a specific tumor subtype. The elucidation of normal lung versus lung tumor gene expression patterns and regulatory targets yields biomarker systems that discriminate lung phenotypes (i.e., biomarkers) and provide a foundation for the discovery of normal and aberrant gene regulatory mechanisms.

**Results:**

We built condition-specific gene co-expression networks (csGCNs) for normal lung, LUAD, and LUSC conditions. Then, we integrated normal lung tissue-specific gene regulatory networks (tsGRNs) to elucidate control-target biomarker systems for normal and cancerous lung tissue. We characterized co-expressed gene edges, possibly under common regulatory control, for relevance in lung cancer.

**Conclusions:**

Our approach demonstrates the ability to elucidate csGCN:tsGRN merged biomarker systems based on gene expression correlation and regulation. The biomarker systems we describe can be used to classify and further describe lung specimens. Our approach is generalizable and can be used to discover and interpret complex gene expression patterns for any condition or species.

**Supplementary Information:**

The online version contains available at 10.1186/s12864-022-08591-9.

## Background

Even though lung cancer incidence has shown a gradual decline in the past decade, it remains the leading cause of cancer death in both men and women with its mortality rate exceeding breast, prostate, colorectal, and brain cancers combined [1]. Lung cancer comprises approximately a quarter of all cancer deaths and is strongly associated with environmental risk factors including smoking and exposure of toxic chemicals that trigger some forms of interstitial lung disease [2]. In 2018, it was estimated that about 2.1 million new people were diagnosed with lung cancer with 1.8 million deaths worldwide [3]. Lung cancer is the leading cause of cancer-related death due to frequent diagnosis at an advanced stage [4]. The early stage of lung cancer patients had 70–90% of 5-year survival rates. However, the patients diagnosed with late stage only had very poor survival [5].

As evidenced by tumor heterogeneity, lung cancer can originate from different tissue contexts, be classified into multiple subtypes, and be present with varied molecular characteristics and biological phenotypes [6]. The most common subtype of lung cancer is non-small cell lung carcinoma (NSCLC), comprising about 85% of all cases. NSCLC can be further divided into major subtypes, including adenocarcinoma (LUAD), squamous cell carcinoma (LUSC), and large cell lung carcinoma [7]. LUAD and LUSC can be distinguished between each other by the complex expression patterns of multiple genes. For example, Charkiewicz et al. identified 53 biomarker genes that can classify LUAD and LUSC with 93% accuracy [8]. Further research by Valeria et al. identified 69 distinct tumor prognostic determinants that had significant impact on clinical factors for LUAD or LUSC, which include key factors on tumor growth, cell cycle, and tumor progression pathways. Those determinants were quite different in LUAD and LUSC, and some of them had opposite impact on these two types of lung cancer [9].

Among candidate lung tumor genes, p63 is the best single marker to separate LUAD from LUSC [10]. Genes related to LUAD are more related to tight junction and cell adhesion molecules, while LUSC related signature genes are more correlated with cell communication pathways [11]. A proper differentiation between lung cancer subtypes at the molecular level is crucial especially for mapping appropriate treatment strategies [12]. For example, the overexpression of epidermal growth factor receptor (EGFR), which is involved in about 60% of NSCLC tumors and present in about 20% of LUAD tumors, currently has precision medicine implications in treating lung cancer [13]. Furthermore, mutations in other genes, such as anaplastic lymphoma kinase (ALK), Kirsten rat sarcoma viral oncogene homolog (KRAS) and ROS proto-oncogene 1 receptor tyrosine kinase (ROS1), can also be factored into targeted therapies [14].

Excellent genomics data repositories exist for discovery of complex gene expression patterns between normal and diseased conditions including transcriptome and DNA polymorphism profiles from The Cancer Genome Atlas (TCGA) and the Genotype-Tissue Expression (GTEx) projects. TCGA is a cancer genomics database which provides a rich amount of high-throughput DNA sequencing and clinical data for different types of cancer based on tissue of origin (portal.gdc.cancer.gov) [15]. TCGA contains both tumor and non-tumor tissue samples excised near the tumor, which are annotated as “solid tissue normal”. GTEx is a public resource database that contains high-throughput data from non-diseased individuals which are collected from 54 non-diseased tissue types for various molecular assays (www.gtexportal.org) [16].

In order to directly compare GTEx and TCGA RNA-seq datasets, Wang et al. developed a RNAseq pipeline to process and unify RNA-seq data from GTEx and TCGA [17]. First, raw sequencing reads were obtained from GTEx and TCGA, re-aligned based on the solid tissue normal samples from TCGA, and re-quantified using RSEM [18]. Finally, batch effects were corrected by running ComBat in the R package SVAseq [19]. This RNAseq pipeline utilized solid tissue normal samples from TCGA to unify data from GTEx and TCGA. Not all TCGA tissue type datasets contain solid tissue normal samples. Thus, only 13 human tissues of origin were unified in total. Of high importance, the unified normal and tumor Gene Expression Matrices (GEMs) built with this pipeline can be processed in the same experiments to identify gene expression shifts between normal and tumor states.

One method to detect condition-specific (i.e. disease) gene expression patterns is gene co-expression network (GCN) analysis, an approach that constructs a gene relationship network where co-expression of genes across multiple samples or specific conditions implies biochemical co-functionality [20, 21]. There are several tools to construct GCNs including weighted correlation network analysis (WGCNA) [22], Bayesian based network construction [23], multiscale embedded gene co-expression network analysis [24], and Knowledge-Independent Network Construction (KINC) [25]. In our study, we utilized KINC 3.4 to construct condition-specific GCNs (csGCNs), which employs Gaussian Mixture Models (GMMs) for clustering for each gene pair to identify gene-gene co-expression clusters that can then be tested for association with experimental conditions such as cancer subtype [26].

While GCNs describe correlated gene expression output, the underlying factors regulating gene output are represented by gene regulatory networks (GRNs). A GRN identifies relationships between regulators and their target genes in a tissue-specific context [27]. In most cases, transcriptional regulation can be determined by the complex interactions among *cis* and *trans* transcription factors (TFs) and their target genes [28]. There are many different approaches to construct GRNs, including several linear models, such as Bayesian network (BN) models [29], dynamic Bayesian network (DBN) models [29], Boolean network [30], and ordinary differential equation (ODE) models [31]. For example, GRNVBEM is an algorithm utilizing Bayesian network [32]; SCODE [33] and GRISLI [34] are the algorithms using linear ODE-based methods. Another method for constructing GRN is based on GCNs [35]. Of relevance to human tumor studies, Sonawane et al. constructed tissue-specific GRNs for 38 human tissues from GTEx in which they combined gene co-expression and protein-protein interaction (PPI) information as well as the DNA motif information together to identify tissue-specific network elements [36]. This study showed that correlated genes were more likely to share a common transcriptional control mechanism [37]. Thus, linking co-expressed genes in either normal condition or disease condition with normal tissue GRNs should be helpful to identify mechanisms underlying diseases, including specific cancer subtypes.

**Fig. 1:**
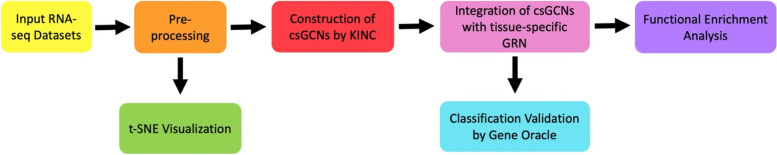
A summary of workflow used for discovering lung tissue specific biomarker systems

The molecular mechanisms underlying complex traits including normal lung development or lung tumor formation are discoverable using systems genetics approaches. In this report, we aimed to discover lung *biomarker systems* which we define as co-functional gene sets that not only discriminate specific conditions or phenotypes (i.e., biomarkers) but also integrates gene regulation information as a foundation for the discovery of genetic control mechanisms between gene expression states. To achieve this goal, we first extracted pairwise gene expression correlations with KINC 3.4 from the unified GTEx-TCGA GEMs to construct normal lung and lung tumor csGCNs. The csGCNs were then combined with a normal lung tissue specific GRN (tsGRN). This integrated gene expression platform enabled the elucidation of candidate control-target biomarker systems for normal and cancerous lung tissue which we will discuss. A summary of this pipeline is shown in Fig. 1. As more condition-specific GEMs and GRNs are reported, our approach will improve the resolution of complex biomarker systems for lung cancer but can be applied more generally to any organ context.

## Results

### Unified normal and tumor sample clustering with t-SNE

The first step to discover biomarker systems based on csGCNs and tsGRNs was to obtain and explore normal and tumor RNA-seq transcriptome profiles. Unified GTEx and TCGA RNA-seq GEM files were obtained from Wang et al. [17]. The profiles that belong to the same tissue of origin were merged into tissue-specific unified GEMs. For example, the GEM files containing GTEx normal lung samples, TCGA solid lung normal samples, TCGA LUAD samples, and TCGA LUSC samples were merged into one lung-specific unified GEM. Each unified GEM for 13 tissues underwent further normalization as described in the Methods section.

**Fig. 2:**
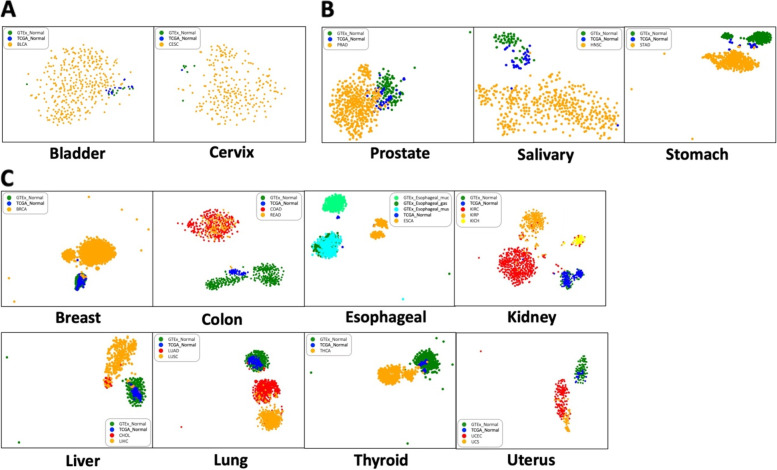
t-SNE visualization of gene expression patterns for 13 unified human normal tissues and tumor subtypes. Each tissue type contains GTEx normal, TCGA solid normal, and at least one tumor subtype. Each color represents a different condition. Cancer samples are labeled as orange, red and yellow; GTEx normal samples are labeled as green, light green and cyan; TCGA solid tissue normal samples are labeled as blue. Samples are separated into multiple clusters based on FPKM RNA-seq gene expression data. **A** t-SNE plots with tissue samples that did not form a cluster. **B** t-SNE plots with TCGA solid tissue normal samples grouped between GTEx normal samples and TCGA tumor samples. **C** t-SNE plots with TCGA solid tissue normal samples grouped together with GTEx normal samples

To further explore the clustering patterns for different tissues, we performed t-distributed stochastic neighbor embedding (t-SNE) [38] visualization (Fig. 2). Some tissue datasets did not form distinct clusters due to the small number of GTEx normal samples or TCGA solid tissue normal samples. For example, the bladder dataset did not show a definitive cluster pattern because there were too few samples for the GTEx normal condition (*n* = 11) and TCGA tumor-flanking “solid tissue” normal condition (*n* = 19) to form clusters. The same situation also occurred for the cervix dataset (Fig. 2A). For those tissue types that did form clusters, some tissue datasets cannot separate TCGA solid tissue normal samples apart from either GTEx normal or TCGA tumor samples. For example, for prostate, salivary, and stomach datasets, the TCGA solid tissue normal samples were located between GTEx normal samples and TCGA tumor samples situation. It was difficult to determine if the TCGA solid tissue normal samples in these organs were truly “normal” samples (Fig. 2B). For the remaining tissue specific datasets, clear patterns of separation between normal samples and tumor samples were visualized. For example, in lung dataset, normal samples, regardless of the source, grouped together as a single cluster and each cancer subtype clustered separately (Fig. 2C). In some tissue datasets, such as stomach, liver, and uterus, the clusters were pushed together aside due to the outlier points, but the overall patterns were still clear. We chose the lung as a target organ to construct condition-specific lung GCNs using KINC followed by integrating the GTEx normal lung GRN with the condition-specific GCN to identify possible regulatory mechanisms in both normal lungs and lung tumors.

### Lung condition-specific gene go-expression network (csGCN) construction

For deeper analysis, we selected the unified and normalized lung GEM that containing 313 GTEx normal lung samples, 110 TCGA solid lung normal samples, 489 adenocarcinoma (LUAD) samples, and 503 squamous cell carcinoma (LUSC) samples to construct a csGCN. The density plot for the normalized lung GEM is shown in Supplemental Fig. 1. The sample stage distribution information is shown in Supplemental Fig. 2, and t-SNE visualization of unified lung samples stage information is shown in Supplemental Fig. 3. Most of the lung cancer patients in TCGA data were in early stage. Few were in stage IV. According to the t-SNE visualization, the stage information of the lung cancer patients cannot separate samples. Using this lung GEM as input, a csGCN was constructed using KINC version 3.4.2. First, KINC identified GMMs and retained any pairwise Spearman correlation greater than |0.5| as a potential edge. KINC then ran a Pearson’s power analysis to remove edges with insufficient power. Next, by providing to KINC the sample condition information, it performed a linear regression test for each edge with each quantitative condition (r-square > 0.3 and *p*-value < 0.001) or two z-score tests of proportions for categorical conditions (*p*-value < 0.001). Edges with association to a condition were labeled with that condition resulting in condition-specific subnetworks. The four conditions for the unified lung GEM include GTEx normal, TCGA normal, LUAD, and LUSC.

There are instances where edge associations can be biased. For example, if expression of one gene is highly variable between conditions, it will bias the pairwise comparison to appear condition-specific even if the other gene is not variable between conditions. Additionally, samples with missing expression in one gene must be removed prior to correlation analysis. If missing values tend to occur in one condition in only one gene, then sample removal will bias the comparison to appear condition specific. To address these issues, KINC next employed a Welch’s one-way ANOVA test (to check for conditional variation in both genes) and a Student’s t-test (to check for equal patterns of missingness) to remove biased edges. Finally, remaining edges were ranked by their correlation value (similarity score), r-square (for quantitative conditions) and *p*-values [26]. All identified edges that were enriched in at least one condition formed the full lung csGCN.

**Fig. 3:**
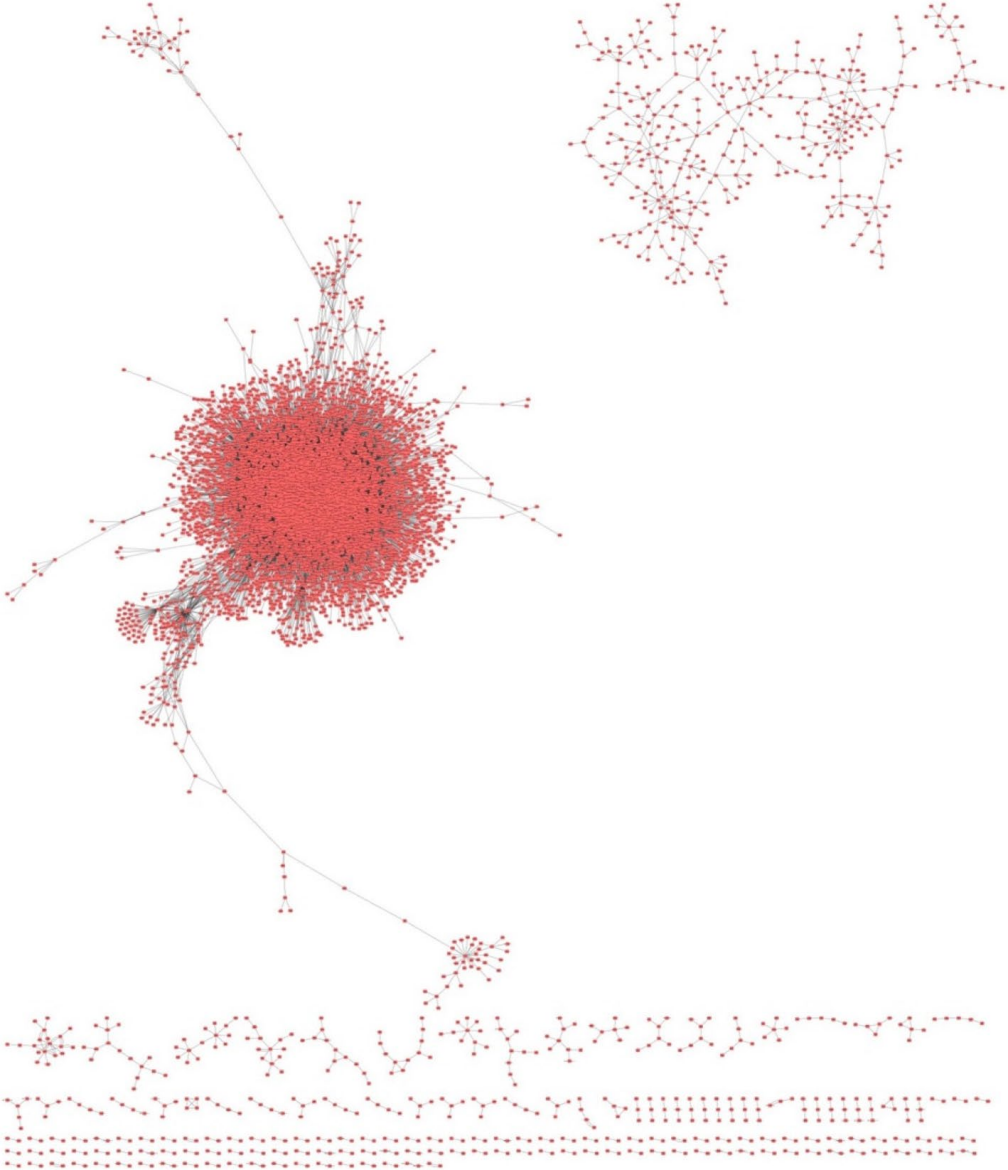
Unified lung condition-specific gene co-expression network (csGCN). The full lung csGCN constructed from 1415 lung RNA-seq samples from 4 different lung conditions: GTEx normal lung, TCGA normal lung, TCGA LUAD tumor, and TCGA LUSC tumors. The GCN is scale-free and contains 7868 nodes and 58,415 edges

The full lung csGCN contained 7868 genes and 58,415 edges, and an average clustering coefficient < C > = 0.281 (Supplemental Table 1; Fig. 3). The global network attributes for both the full network and each condition-specific sub-networks are shown in Table 1. Connectivity, clustering coefficient, unique edge percentage, and unique node percentage for each csGCN were calculated (Table 1). The clustering coefficient is the measure of the overall tendency of nodes to form clusters or groups. For the module-free scale-free network, <C > is usually very low [39]. The GTEx normal csGCN contained the most nodes and edges, and the highest average clustering coefficient (6813 nodes, 53,233 edges, and < C > =0.291). The TCGA normal subnetwork was small and contained had the lowest average clustering coefficient (36 nodes, 21 edges, and < C > =0). The TCGA normal subnetwork also had the least average connectivity (1.17), least unique edge percentage (1.53%), and least unique node percentage (3.4%). The LUAD specific subnetwork contained 530 nodes, 600 edges, and < C > =0.002. The LUSC specific subnetwork contained 1414 nodes, 1694 edges and < C > of 0.062. A 3D network visualization of lung GCN is shown in Supplemental Fig. 4 where one can observe that the four csGCN subnetworks can be separated in four sub-clusters.

**Table 1:** Lung Condition-Specific Gene Co-Expression Network Global Attributes

Sample Condition	Samples	Nodes	Edges	Connectivity	Unique Nodes	Unique Edges	Connectivity	Unique Edge	Unique Node	Clustering
								Percentage	Percentage	Coefficient
*Full Network*	1415	7868	58,415	14.85	–	–	–	–	–	0.281
*GTEx_NORMAL*	313	6957	54,664	15.71	6813	53,233	15.63	97.38	97.93	0.291
*TCGA_NORMAL*	110	1059	1370	2.59	36	21	1.17	1.53	3.40	0.000
*TCGA_LUAD*	489	542	652	2.41	530	600	2.26	92.02	97.79	0.002
*TCGA_LUSC*	503	1429	1729	2.42	1414	1694	2.40	97.98	98.95	0.062

### Condition-specific lung GCN and GRN integration

The GTEx normal lung specific gene regulatory network (GRN) was downloaded from Zenodo https://zenodo.org/record/838734 [36]. The entire GTEx normal lung related GRN is shown in Supplemental Table 2. We then integrated the normal lung tsGRN with our csGCNs. Edges in the normal lung GRN were selected where the TFs targeted at least one node in LUAD and LUSC specific GCNs to obtain LUAD and LUSC GRN networks.

DEG analysis was then performed for each gene in the unified GEMs to identify differentially expressed genes (DEGs) between GTEx normal lung and TCGA LUAD conditions as well as GTEx normal lung and TCGA LUSC conditions. For both LUAD and LUSC conditions, significant DEGs were determined for both a transcription factor (TF) and its target gene (TR). TF or TR genes were considered as DEGs when their DEseq2 adjusted *p*-value was less than 0.001; gene expression directionality (e.g., up−/down-regulation in tumor) was noted.

**Fig. 4:**
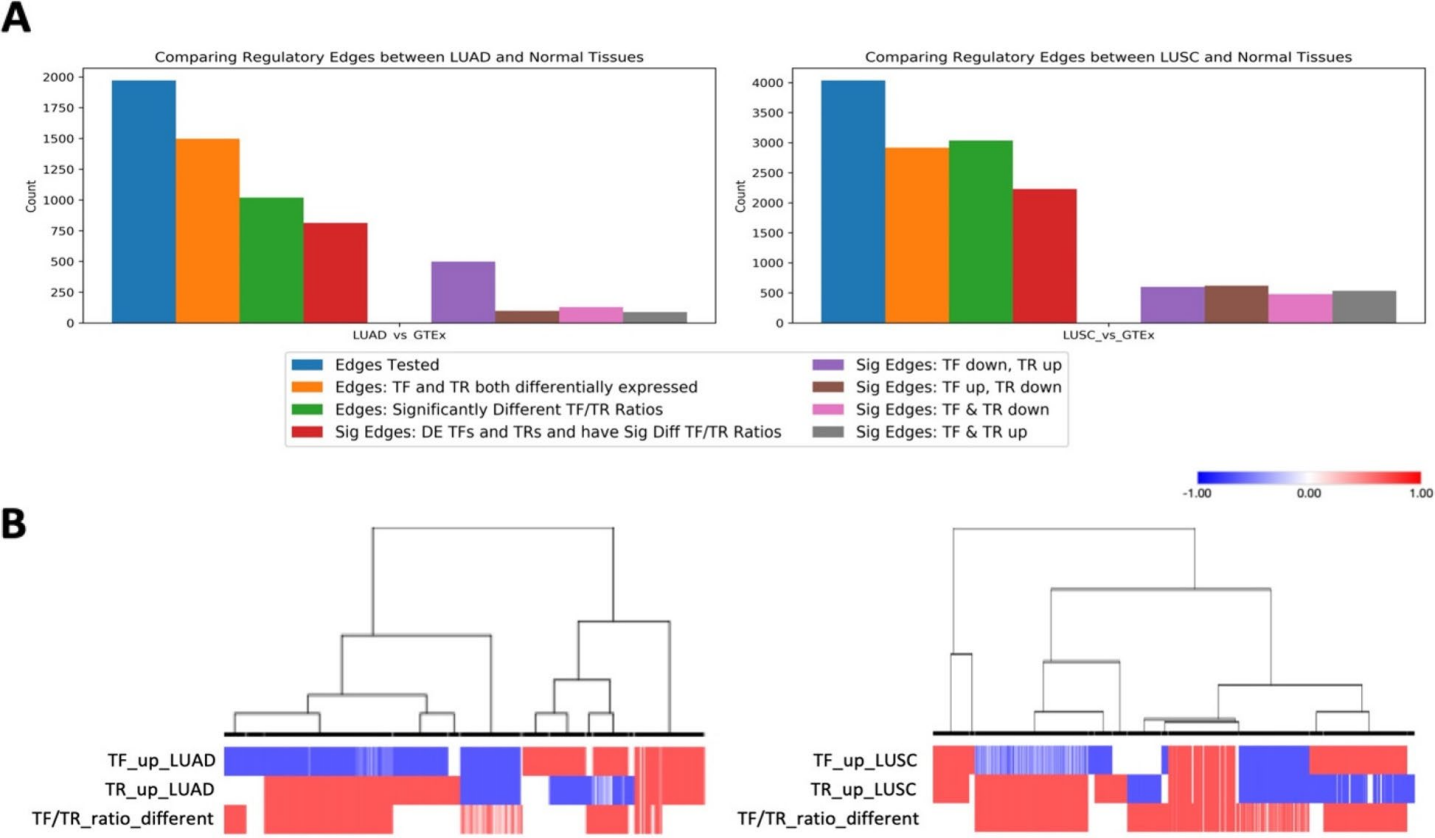
Summary of regulatory edges between GTEx normal lung and two lung cancer subtypes. **A** Bar graphs calculating number of GRN edges between GTEx normal lung tissue samples and LUAD (left panel) or LUSC (right panel) respectively for each category. Blue bars represent the number of regulatory edges in total where TF regulated at least one node in an edge from the LUAD/LUSC specific GCNs. Orange bars represent the number of edges when both TF and TR were differentially expressed. Green bars represent the number of edges when TF/TR ratio was significantly different between normal lung and LUAD or LUSC. Red bars represent the number of significant edges when TF and TR were both DEGs and TF/TR ratio is significantly different. For those significant edges, purple bars represent TF was down-regulated in cancer while TR was up-regulated in cancer; brown bars represent TF was up-regulated in cancer while TR was down-regulated in cancer; pink bars represent both TF and TR were down-regulated; and bars represent both TF and TR were up-regulated. **B** Heatmap distribution of regulatory edges comparing between normal samples and two types of lung cancer with LUAD on the left and LUSC on the right. For DEGs up/down regulation, red means up-regulation in lung cancer, blue means down regulation in lung cancer, and white means not a DEG. For TF/TR ratio, red means the ratio was significantly different between normal and cancer, and grey means the ratio is not different

For each edge pair, the ratio of the expression value of TF and TR genes (TF/TR ratio) was calculated respectively for samples from GTEx normal, LUAD, and LUSC conditions. A Student’s t-test was performed (*p* < 0.001) to determine if TF/TR ratio of each edge pair was significantly different between GTEx normal lung and LUAD or LUSC conditions. DEGs for TF and TR meant those genes were significantly different between normal and cancer condition, and the TF/TR ratio differences meant the edges were different between normal and cancer condition. The counts of each edge category and distribution heatmap comparing regulatory edges from GTEx normal lung samples with different types of lung cancer samples are shown in Fig. 4. The detailed DEG results and TF/TR ratio comparison for condition specific GRN edges are shown in Supplemental Table 3. As seen in Fig. 4 and Table 2, there were 1972 regulatory edges in total for nodes in the LUAD specific csGCN. Among those edges, 1497 edges contained both TF and TR that were DEGs between GTEx normal lung and LUAD samples, 1019 edges contained TF/TR ratio that was significantly different between GTEx normal lung and LUAD conditions, and 813 significant edges were DEGs and significantly different TF/TR ratios. For the significant edges containing both situations, most (498) showed that the TFs were down-regulated in LUAD versus normal while TRs were up-regulated, which means the downregulation of the TFs could result in the up-regulation of the corresponding target genes that play roles in LUAD cancer development. The number of edges with significant TF/TR ratios was similar for TF up/TR down, both TF down/TR down, and TF up/TR up patterns (98, 128, and 89 respectively).

**Table 2:** Lung Condition-Specific Regulatory Edges Comparison

Comparison	Total Edges	DEseq2*	TR/TF*	DEseq2* + TR/TF*	TF & TR Up*	TF & TR Down*	TF Up & TR Down*	TF Down & TR Up*
LUAD_vs_GTEx	1972	1497	1019	813	89	128	98	498
LUSC_vs_GTEx	4037	2917	3036	2229	533	479	618	599

*Up* Up in tumor, *Down* down in tumor

*Significance was adj. *p* < 0.001 for DESeq2 and *p* < 0.001 for TR/TF ratio test

There were 4037 regulatory edges in total with TFs targeting at least one node in the LUSC csGCN. Among those edges, 2229 were identified as significant edges as both TFs and TRs were DEGs and the TF/TR ratio was significantly different between normal to LUSC. The number of significant edges for all four conditions were similar (Fig. 4A and Table 2). The heatmap distribution was also performed for those edges. For the GRN edges comparing GTEx normal and LUAD conditions, there were more down-regulated TFs in LUAD than up-regulated TFs, but more up-regulated target genes. Thus, the TF down/TR up pattern contains most significant edges. However, the up or down regulation pattern was split in half for both TF and TR in GRN edges comparing GTEx normal and LUSC, so the significant edges showed similar number for each of the four conditions (Fig. 4B).

**Fig. 5:**
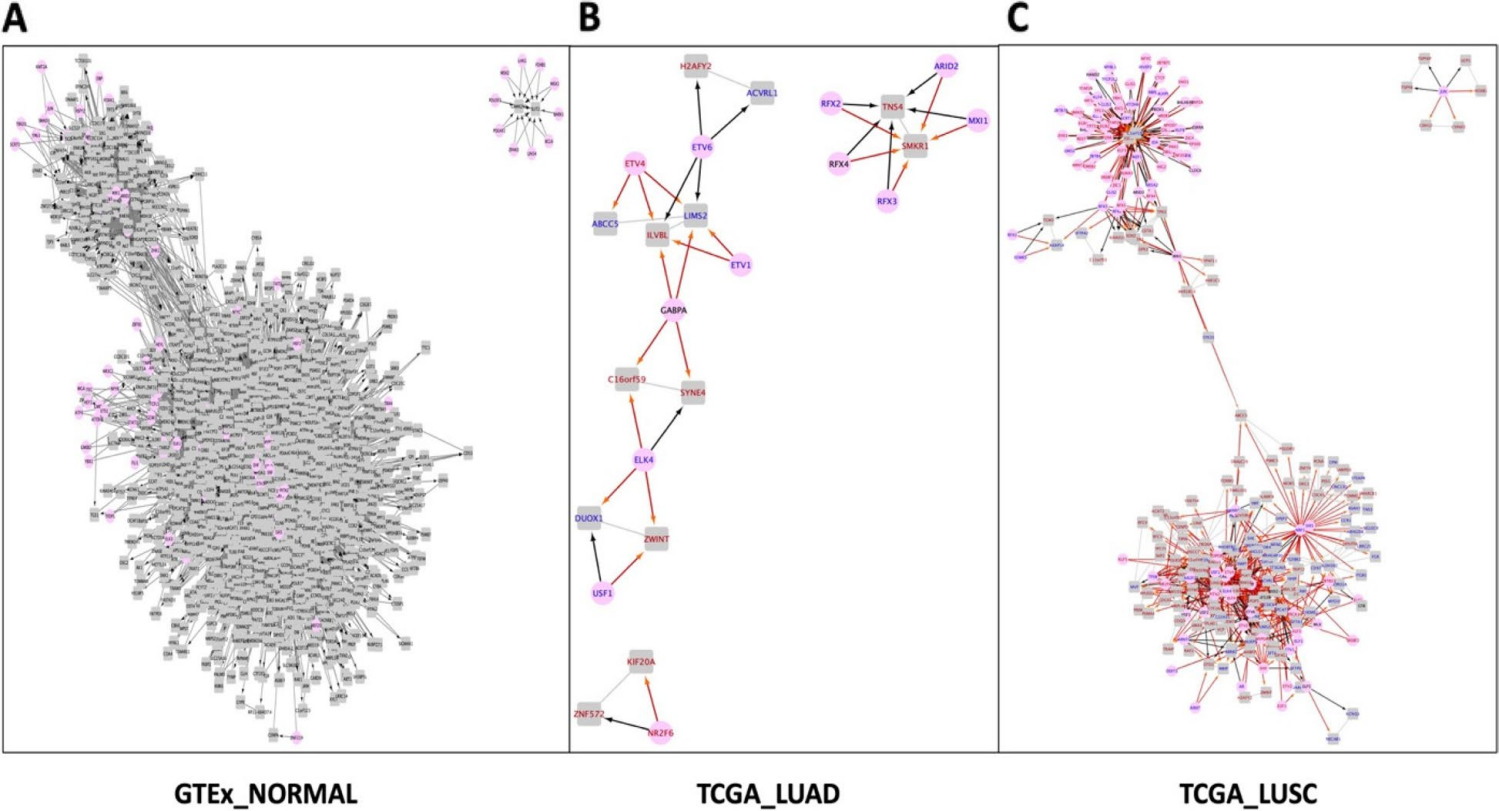
The merged triangle network of TFs from GRNs regulating both nodes in csGCN subnetwork edges. **A** The merged network for GTEx normal lung subnetwork mapped with GRN. **B** The merged network for TCGA LUAD subnetwork mapped with GRN. **C** The merged network for TCGA LUSC subnetwork mapped with GRN. Pink round nodes represent TFs, grey rectangular nodes represent genes that are both regulated by TFs and exist in each csGCN subnetworks. A line with an arrow indicates a directed edge and the line without arrow is the undirected edge. For lung cancer specific sub-networks, the name of up-regulated DEGs are shown in red; the name of down-regulated DEGs are shown in blue; non-DEGs were shown in black. GCN undirected edges are shown in grey. The directed edges when TF/TR ratio was different from normal to cancer are shown in orange, while directed edges when TF/TR ratio was not significantly different are shown as black

To identify gene pairs potentially controlled by common regulatory factors, we selected csGCN edges where both nodes are the target of the same TF forming a triangle network motif. The merged csGCN and GRN node and edge for LUAD and LUSC specific networks are shown in Fig. 5 and a Cytoscape network file can be found in Supplemental Data. Each TF that can regulate both nodes of a csGCN edge in lung tissue forms a triangle. Gene names in red represented up-regulation in cancer, gene names in blue represented down-regulation in cancer, and gene names in black represented non-DEG genes. For the edge attribute, if the TF/TR ratio was significantly different between GTEx and lung cancer, the directed edge color was orange. The detailed triangle edge information is shown in Supplemental Table 4.

Four triangles were found in LUAD, such that the TF targeting both nodes in a LUAD specific GCN edge. For example, all three edges in triangle of ETV4 targeting both ABCC5 and LIMS2 in the LUAD csGCN were DEGs. The up-regulation of ETV4 in tumors is associated with down-regulation of both ABCC5 and LIMS2 in tumors. Further, both the ETV4/ABCC5 ETV4/LIMS2 ratios were both significantly different between GTEx normal condition and LUAD. In LUSC, 169 triangles were found that the TFs pointing to both nodes in a LUSC specific csGCN edge were significant GRN edges.

### Biomarker system validation

**Fig. 6:**
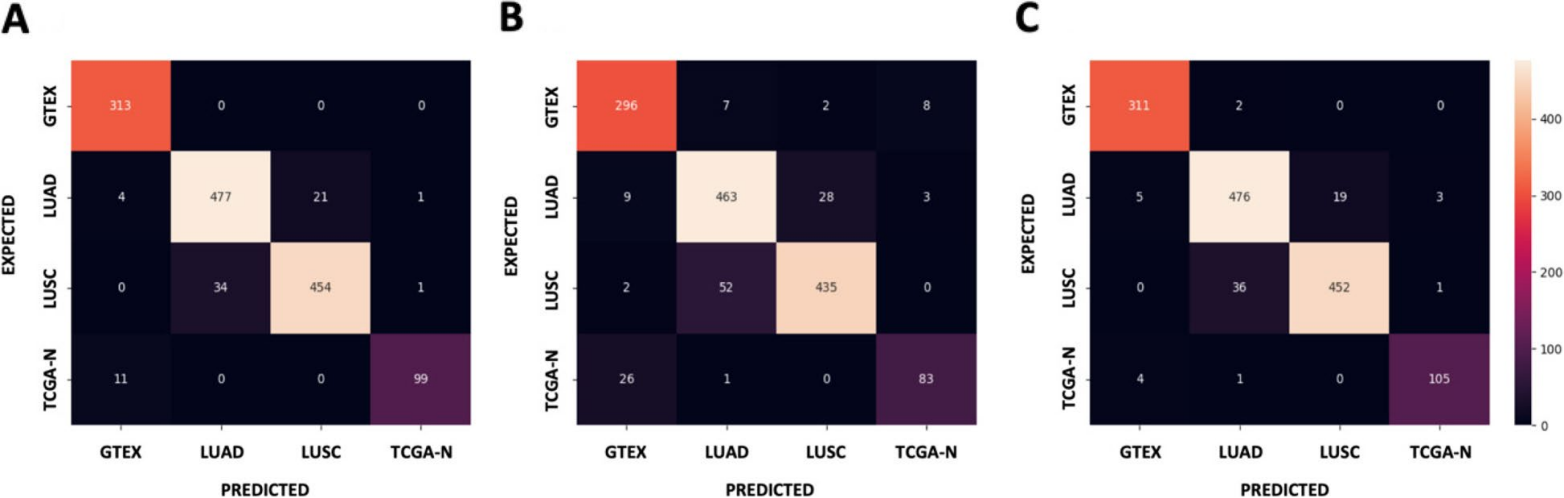
Gene Oracle classification confusion matrices from “triangle” csGCN nodes. The number in the diagonal boxes indicates the number of samples that are correctly classified, and other boxes show the number of misclassified samples. **A** The confusion matrix for GTEx normal gene sets. **B** The confusion matrix for TCGA LUAD gene sets. **C** The confusion matrix for TCGA LUSC gene sets

In order to test the classification potential of our selected genes, a deep learning algorithm called Gene Oracle performed sample classification according to input gene expression patterns [40]. Gene Oracle utilized a multilayer perceptron (MLP) neural network to measure the classification. The MLP consists of an input GEM layer, three hidden layers, and a final softmax layer for classification. 70% of the dataset was trained and the rest 30% of the dataset was then evaluated. The separation of test and trained datasets was randomly determined. The input data we used for Gene Oracle were subsets of gene expression matrices collected from the normalized unified lung FPKM GEM. The FPKM GEM contains 19,648 genes. We selected genes and TFs showed in the merged triangle network for GTEx normal, TCGA LUAD, and TCGA LUSC conditions, respectively, as condition-specific gene sets. Then we generated subGEMs by mapping those gene sets to the normalized lung GEM. The three subGEMs were the input for Gene Oracle to evaluate the cancer-type classification accuracy of samples. The confusion matrix for each gene set analyzed is shown in Fig. 6 where the number of correctly classified samples are shown in the diagonal boxes.

Most samples were classified correctly using the condition-specific gene expression profile. Based on the gene expression of GTEx normal specific 1459 genes, all GTEx normal samples were correctly classified, while other groups had some mis-classified samples. For both LUAD specific expression profile (13 genes) and LUSC specific gene expression profile (150 genes), most samples were correctly classified. The TCGA normal condition also had several mis-classified as GTEx normal which is understandable given that they are both considered to be normal lung samples as further evidenced by the t-SNE plot in Fig. 2.

### Biomarker system functional enrichment analysis

By integrating the lung specific tsGRN network with csGCNs, we found specific biomarker systems that might be involved in LUAD and LUSC tumor biology. Functional enrichment analysis was performed on these gene sets with ToppFun (https://toppgene.cchmc.org/enrichment.jsp) using TFs and their target genes from each triangle network as the input gene list. The annotation databases we tested included REACTOME [41], KEGG [42], and Pathway Interaction Database (PID) [43]. The ToppFun enriched pathways can be found in Supplemental Table 5.

In the LUAD csGCN, four TFs out of 180 were associated with the “transcriptional mis-regulation in cancer” pathway (Bonferroni *p* = 7.4E-3). These TFs were ETV1, ETV4, ETV6 and ELK4. For selected TFs which targeted edges from TCGA LUAD network, the edge of ILVBL and LIMS2 was regulated by ETV1, ETV4 and ETV6 simultaneously. The edge of LIMS2 and ABCC5 was regulated by ETV4. LIMS2 had many edges in GTEx normal csGCN, while both ILVBL and ABCC5 genes cannot be found in any nodes of the GTEx normal csGCN. Both ILVBL and ABCC5 genes had higher percentage of somatic mutations in LUSC relative to LUAD cases (Table 3).

**Table 3:** Genes Selected for Deeper Analysis

Gene	Description	Condition	LUAD mutation rate^**a**^	LUSC mutation rate^**a**^
ETV1	ETS Variant Transcription Factor 1	LUAD	1.23%	1.82%
ETV4	ETS Variant Transcription Factor 4	LUAD	0.35%	0.61%
ETV6	ETS Variant Transcription Factor 6	LUAD	1.06%	2.63%
ELK4	ETS Transcription Factor ELK4	LUAD	0.88%	0.40%
LIMS2	LIM and senescent cell antigen-like domains 2	LUAD	0.71%	1.62%
ILVBL	IlvB Acetolactate Synthase Like Protein	LUAD	0.71%	1.41%
ABCC5	ATP Binding Cassette Subfamily C Member 5	LUAD	3.88%	4.65%
PSMA4	proteasome 20S subunit alpha 4	LUSC	0.71%	1.21%
PSMC5	proteasome 26S subunit, ATPase 5	LUSC	0.88%	0.61%
E2F1	E2F transcription factor 1	LUSC	0.53%	0.81%
MCM5	Minichromosome Maintenance Complex Component 5	LUSC	2.65%	2.63%
GINS1	GINS Complex Subunit 1	LUSC	0.35%	0.81%
GINS2	GINS Complex Subunit 2	LUSC	0.35%	–
CDC45	Cell Division Cycle 45	LUSC	0.71%	1.62%
RFC4	Replication Factor C Subunit 4	LUSC	1.41%	2.42%
RFC5	Replication Factor C Subunit 5	LUSC	0.88%	1.21%
PRIM1	DNA Primase Subunit 1	LUSC	0.53%	2.02%
PCNA	Proliferating Cell Nuclear Antigen	LUSC	0.18%	0.61%
SPC25	SPC25 Component Of NDC80 Kinetochore Complex	LUSC	0.53%	0.20%
SFTA3	Surfactant Associated 3	LUSC	0.71%	0.61%
SFTPA2	Surfactant Protein A2	LUSC	0.53%	2.22%
SFTPB	Surfactant Protein B	LUSC	1.59%	1.21%
SFTPD	Surfactant Protein D	LUSC	0.71%	1.01%

^a^ Mutation rates are the percent cases with simple somatic mutations on the target gene

For the LUSC csGCN, several genes were found related to DNA replication. The pathway called “DNA replication” contained eleven out of 111 genes, including PSMA4, PSMC5, E2F1, MCM5, GINS1, GINS2, CDC45, RFC4, RFC5, PRIM1, and PCNA. Several edges can be found for those genes in the LUSC csGCN. For example, CDC45 forms edge with MAM5 and PCNA. Also, several of these eleven genes formed edges with same node. For example, SPC25 formed edges with RFC5 and PRIM1. The expression pattern of these genes showed that most had differential expression between normal and LUSC. Another pathway we identified was related to defective CSF2RA which causes pulmonary surfactant metabolism dysfunction 5 (SMDP5). Four out of eight genes were found, including SFTA3, SFTPA2, SFTPB, and SFTPD. It has been shown that a rare missense mutation in SFTPA2 can cause idiopathic pulmonary fibrosis and lung cancer [44]. These genes were all down-regulated in lung cancer and showed more down-regulation in LUSC than LUAD samples. There were no shared GCN edges between these four genes, but they do share same TFs. Thus, the edges from LUSC specific GCN containing these genes could be involved in this pathway and had function in forming LUSC.

We identified another four out of eight genes in the pathway called “defective CSF2RA causes pulmonary surfactant metabolism dysfunction 5 (SMDP5)” (Bonferroni *p* = 5.73E-3). Most selected genes from LUSC networks had higher mutation rates in LUSC than LUAD. Among those genes, SFTPA2 had much higher mutation rate in LUSC than LUAD. The expression pattern of the selected genes is shown in Table 4.

**Table 4:** Gene Expression Patterns of Selected Genes

Genes	Condition	log2Fold	*p-value	DEG	Up in LUAD/LUSC
ETV1	LUAD	0.59	2.82E-15	Y	N
ETV4	LUAD	−3.71	0.00E+ 00	Y	Y
ETV6	LUAD	0.17	1.26E-04	Y	N
ELK4	LUAD	0.39	1.20E-25	Y	N
LIMS2	LUAD	3.66	0.00E+ 00	Y	N
ILVBL	LUAD	−0.28	1.02E-11	Y	Y
ABCC5	LUAD	0.52	3.04E-19	Y	N
PSMA4	LUSC	−0.93	3.90E-181	Y	Y
PSMC5	LUSC	−0.38	1.28E-43	Y	Y
E2F1	LUSC	−1.81	2.47E-184	Y	Y
MCM5	LUSC	−1.82	0.00E+ 00	Y	Y
GINS1	LUSC	−4.35	0.00E+ 00	Y	Y
GINS2	LUSC	−4.03	0.00E+ 00	Y	Y
CDC45	LUSC	−4.10	0.00E+ 00	Y	Y
RFC4	LUSC	−2.92	0.00E+ 00	Y	Y
RFC5	LUSC	−1.92	0.00E+ 00	Y	Y
PRIM1	LUSC	−2.00	0.00E+ 00	Y	Y
PCNA	LUSC	−2.33	0.00E+ 00	Y	Y
SPC25	LUSC	−3.36	0.00E+ 00	Y	Y
SFTA3	LUSC	2.97	4.41E-87	Y	N
SFTPA2	LUSC	2.25	2.01E-38	Y	N
SFTPB	LUSC	2.64	2.43E-68	Y	N
SFTPD	LUSC	1.94	3.61E-41	Y	N

*LUAD* lung adenocarcinoma, *LUSC* lung squamous cell carcinoma

*Adjusted *p* values less than 1e-03 represents this gene was differentially expressed between GTEx normal and LUAD or LUSC

## Discussion

Lung cancer is a highly complex disease. The subsets of lung tumors show diverse patterns of gene expression. In this study, lung csGCNs were generated and were compared with normal lung specific tsGRNs. The number of edges and nodes enriched in TCGA LUSC csGCN was approximately three times those in the TCGA LUAD csGCN, even though the sample size was similar in the two conditions. Many unique edges were found in LUAD and LUSC csGCNs, which indicated that the two lung cancer subtypes may have distinct tumor gene expression profiles.

Many genes in the LUAD and LUSC csGCNs are known to be involved in cancer. For instance, many prognostic gene determinants identified by Relli et al. [9], which showed significantly different survival impacts on LUAD and LUSC patients, can be found in the LUAD and LUSC csGCNs respectively. For example, many LUAD associated genes, such as FOLR1, SFTA3, TMC5, and TMEM125, can be found in the LUAD csGCN network. Furthermore, determinants showing negative prognostic impact on LUAD, but positive impact on LUSC, such as DSG3, FOXE1, GRHL3, DLX5, and TMPRSS11D, can be found in the LUSC csGCN. Yao et al. identified prognostic biomarkers in LUAD, which contains 12 lncRNAs, nine mRNAs and one miRNA that were significantly (*p* < 0.001) associated with the overall survival with LUAD patients. Five out of nine mRNAs were identified in the LUAD csGCN, including CCNE1, CCNB1, KIF23, CEP55, and CHEK1 [45]. Similarly, Dong et al. constructed lncRNA-miRNA-ceRNA network that revealed pathological roles of the LUAD and LUSC. Only two of twenty mRNAs in LUAD that were also identified by our LUAD specific GCN (UBE2C and CTHRC1), while nine out of twenty mRNAs were identified in LUSC specific GCN, including SFTPA2, CLDN18, SFTPB, SFTPD, NAPSA, CALML3, SPRR1B, KRT6B, and KRT5 [46].

According to the merged tsGRN-csGCN network, we can tell that each targeted gene can be regulated by multiple transcription factors, and each transcription factor can regulate a lot of genes as well. Thus, even merge the regulation relationships with the correlation networks in each condition, we still cannot figure out what is the potential reason for edges being altered from normal condition to cancer. Evidence shows that genes with high correlation and with similar functions are more likely to be regulated by the same mechanism [37]. Highly co-expressed genes are more likely to share same TFBS and thus regulated by same transcription factor. By extracting triangle network motifs that TFs regulating both nodes in the csGCN subnetwork edges, we can further investigate the regulatory mechanisms underneath the alteration in gene co-expression relationships for different conditions.

By performing functional enrichment on genes and TFs in LUAD and LUSC csGCNs, we found several genes and TFs participate in the same biological pathway. For the LUAD network, four TFs, ETV1, ETV4, ETV6, and ELK4, were involved in the pathway called “transcriptional mis-regulation in cancer”. All four TFs were normal lung and LUAD DEGs. The edge (LIMS2, ILVBL) is potentially regulated by ETV1, ETV4, and ETV6 simultaneously, and LIMS2 was also co-expressed with ABCC5, which were both regulated by ETV4. The three target nodes were all involved in our LUAD specific significant triangles.

We searched these three target nodes in all csGCNs. Both ILVBL and ABCC5 genes did not form any edges in GTEx normal condition, which indicated that gaining the (ILVBL, LIMS2), (ILVBL, LIMS2), and (LIMS2, ABCC5) edges could be related to the formation of LUAD cancer. Both LIMS2 and ABCC5 were down-regulated in LUAD, while ILVBL was up-regulated in LUAD. Chang et al. found that up-regulation of ETV4 resulted in the up-regulation of MSI2 in LUAD, which promotes proliferation and invasion of LUAD [47]. Our results suggest that the up-regulation of ETV4 can both down-regulate ABCC5 and LIMS2 and up-regulate ILVBL, which may also result in the proliferation of LUAD. Thus, our merged csGCN-tsGRN network especially for LUAD and LUSC could give us potential regulation information in forming different types of lung cancer.

Many studies have previously described the role four of the TFs we identified in non-small cell lung cancer. Zhang et al. identified ETV1 is one of the potential oncogenic TFs that are critical to non-small cell lung cancer [48]. Wang et al. found that overexpression of ETV4 upregulated PXN and MMP1 that promotes progression of non-small cell lung cancer [49]. PXN was found in our LUSC specific GCN, and MMP1 was found in our LUAD specific GCN. Liang et al. studied the expression pattern of ETV6/TEL related to non-small cell lung cancer patients on survival [50]. Kossenkov et al. found the binding sites for ELK4 was enriched in the promoter regions of genes which are up-regulated in tumor [51]. For the target genes, only ABCC5 was identified to have function on gemcitabine sensitivity that related to non-small cell lung cancer [52]. Our study suggests that the regulatory changes for ABCC5 and LIMS2 led to the correlation of these two genes only existed in LUAD, which could be associated with LUAD cancer etiology.

The tsGRN was generated from GTEx normal samples. The regulatory information for TCGA tumor datasets cannot be found. Some transcriptional regulation might be extensively changed in cancer, which will result in new regulatory edges that are not present in normal GRN. Thus, some transcriptional factors cannot be detected due to this reason. This is one of the limitations of our analysis. The reason why we integrated tissue-specific GRN with condition-specific GCNs is that we want to further investigate how those correlated genes disappeared or showed in different types of lung cancer compared to normal condition. Even though the tissue specific GRN was generated from GTEx normal lung samples and might miss some of the TF links, this integration still gave us some hint of why correlated genes altered from normal condition to different types of cancer.

## Conclusions

The utility of biomarkers in lung cancer helps in early detection, prognosis, and treatment guidelines, especially helpful for different subtypes of lung cancer. Our study describes how regulatory-linked biomarker systems can be discovered in different types of lung cancers using csGCN analysis and integration with tsGRNs. In future studies, stage information can be considered, and our approach can be used to interpret complex gene expression patterns between metastatic and non-metastatic lung cancer samples as well as other types of tumors.

## Materials and methods

### Input data and gene expression matrix (GEM) preparation

All available gene expression FPKM files for GTEx normal samples, TCGA solid normal samples, TCGA tumor samples of each tissue type were downloaded from the data records of Wang’s research [10.6084/m9.figshare.5330593] [17]. All files were quantile normalized and corrected for batch effects. For each tissue type, we merged those GTEx normal, TCGA solid normal and TCGA tumor files together into one GEM using GEMprep [https://github.com/SystemsGenetics/GEMprep.git]. The condition-specific sample annotation matrix was collected from the original GEMs of each condition. The merged GEM then underwent the log2 transformation, quantile normalization, and Kolmogorov-Smirnov test (KS Dval> 0.15) by using the normalization function in GEMprep. No outlier sample was removed by the KS test analysis for each tissue. For unified lung dataset, the total of 1415 samples were downloaded, including 313 GTEx normal lung samples, 110 TCGA normal samples, 489 TCGA LUAD tumor samples, and 503 TCGA LUSC tumor samples. Each file contains the measurements of 19,648 genes. The density plot for unified lung GEM, which is the gene expression distribution of each sample, is shown in Supplemental Fig. 1.

### Gene co-expression network construction

The Knowledge Independent Network Construction (KINC) software (https://github.com/SystemsGenetics/KINC) was used to identify gene correlation relationships from the gene expression data. KINC was performed on an NVIDIA DGX-2 workstation. KINC 3.4.2 was pulled in the Docker environment. The network construction used Gaussian Mixture Models (GMMs) to identify clusters before calculating correlation for each cluster, for each gene pair. Only clusters with equal to or greater than 30 samples underwent Spearman correlation and up to five clusters could be detected. The number of identified clusters was between one and five. All log2 transformed and normalized FPKM expression values less than 0 and more than 15 were ignored. We retained all gene pairs with a Spearman correlation value greater than 0.5 or less than − 0.5. Because we used a very low minimum similarity score threshold, we found many potential edges. A Pearson’s power analysis test for the GMM method was performed to filter the low powered clusters using the pwr.r function in the pwr R package. Alpha setting limited the Type I error to the significance of 0.001, and power setting allows 20% Type II error. The condition (GTEX, TCGA_NORMAL, LUAD, LUSC) specificity test was performed to generate condition-specific subnetworks. Linear regression for quantitative conditions and two z-tests for proportions of categorical data was performed. For linear regression test, both r-square values and *p*-values were calculated to obtain condition-specific edges. R-square value counts for the variation of the trend line, and significant p-value indicates how well the samples in the cluster showed the correlation pattern. We set the r-square value to greater than 0.30 and p-value less than 0.001. After extraction of the condition-specific networks, two cases of biased condition-specific edges were removed, including lack of differential cluster expression (DCE) and unbalanced missing data, by using KINC.R package (https://github.com/SystemsGenetics/KINC.R). The package used a Welch’s one-way ANOVA test to identify DCE and a Student’s t-test to compare missing data. Edges without significant p-values for both tests (a *p*-value more than 0.001 for Welch’s ANOVA test and a p-value less than 0.1 for Student’s t-test) were removed. A series of summary plots were generated to check condition-specific response in the network. The last filtering step was to rank the network based on the correlation value (similarity score), the r-square value (for quantitative conditions) and corresponding p-value also by using KINC.R package. Rank was performed for our condition-specific networks, but no edges were removed. This ranking method helped prioritize higher ranked edges. The final step was to visualize the whole network using cystoscope as well as the KINC 3D network viewer [26]. The 3D layout screenshot was shown in Supplemental Fig. 4. The full GCN is shown in Supplemental Table 1.

### Network integration and analysis

The GTEx GRNs were generated by Sonawane et al. (https://sites.google.com/a/channing.harvard.edu/kimberlyglass/tools/gtex-networks) [36]. Some GRN edges were only found in lung tissue, while some other edges were enriched in several tissue types but were still considered as tissue-specific edges. The full GRN network is shown in Supplemental Table 2. The lung condition-specific GCN subnetworks were mapped to the GTEx normal lung GRN network. We selected the TFs that can regulate at least one nodes of the edge as well as both nodes of the edge from each condition-specific subnetwork. DEG analysis was performed between GTEx normal lung GEM and LUAD as well as GTEx normal lung GEM and LUSC by using DESeq2_1.30.1 in R 4.0 (https://bioconductor.org/packages/release/bioc/html/DESeq2.html). The input lung GEM was obtained from 10.6084/m9.figshare.5330539. For each comparison, we determined the TF/TR ratio value, mean and standard deviation in each sample condition and performed a Student’s t-test to determine if a given ratio was significantly different from normal to cancer conditions (*p* < 0.001). All ratio comparisons are shown in Supplemental Table 3. We also determined which TFs and TRs were up-regulated or down-regulated using DESeq2 results. Group classification was performed on condition-specific gene sets using the deep learning software, Gene Oracle [40] (https://github.com/SystemsGenetics/gene-oracle). The MLP model contained five layers: an input layer with the size of gene set, three hidden layers of 512, 256 and 128 units using rectified linear unit (ReLU) activation function [53], and a final later for classification. Confusion matrices were generated using Gene Oracle. Functional enrichment analysis was performed using all genes and TFs in LUAD and LUSC combined networks with Toppfun (https://toppgene.cchmc.org/). We focused on the pathway results. Genes shown in the same pathway, as well as the target genes regulated by TFs in the same pathway, were selected, aiming to find any candidate genes associated with specific lung cancer.

## Supplementary Information

**Additional file 1: Table S1.** Unified Lung Gene Co-expression Network. **Table S2.** GTEx Lung Gene Regulatory Network. **Table S3.** LUAD and LUSC Specific GRN Edge Attributes. **Table S4.** All Triangle Edges Information for LUAD and LUSC Specific Nodes in Combined Networks. **Table S5.** Pathway Information for LUAD and LUSC Specific Nodes in Combined Networks.

**Additional file 2: Figure S1.** Unified Lung Matrix Density Plot. **Figure S2.** Unified Lung Sample Stage Distribution. **Figure S3.** t-SNE Visualization of Gene Expression Patterns for Unified Lung Samples Stage Information. **Figure S4.** Lung GCN 3D Network Visualization.

**Additional file 3:.** Cytoscape file.

## Data Availability

Unified gene expression FPKM files were downloaded from 10.6084/m9.figshare.5330593. GRN networks were downloaded from https://sites.google.com/a/channing.harvard.edu/kimberlyglass/tools/gtex-networks.
