# Supplementary material for: Identification of condition-specific regulatory mechanisms in normal and cancerous human lung tissue
Source: BMC Genomics. 2022 May 6;23:350. doi: 10.1186/s12864-022-08591-9 (PMC9077899; doi:10.1186/s12864-022-08591-9)
Supplement: Supplementary file 2 — Additional file 2: Figure S1. Unified Lung Matrix Density Plot. Figure S2. Unified Lung Sample Stage Distribution. Figure S3. t-SNE Visualization of Gene Expression Patterns for Unified Lung Samples Stage Information. Figure S4. Lung GCN 3D Network Visualization. [file 12864_2022_8591_MOESM2_ESM.docx]

**Supplementary Figures**

**Identification of condition-specific regulatory mechanisms in normal and cancerous human lung tissue**

Yuqing Hang^1^, Josh Burns^2^, Benjamin T. Shealy^3^, Stephen P. Ficklin^2^, and Frank A. Feltus^1,4,5^

^1^Clemson University Department of Genetics & Biochemistry 29634 USA

^2^Washington State University, Department of Horticulture 99164 USA

^3^Clemson University Department of Electrical and Computer Engineering 29634 USA

^4^Clemson University Biomedical Data Science and Informatics Program 29634 USA

^5^Clemson University Center for Human Genetics 29634 USA

*Corresponding Author. Email: [ffeltus@clemson.edu](mailto:ffeltus@clemson.edu)

**
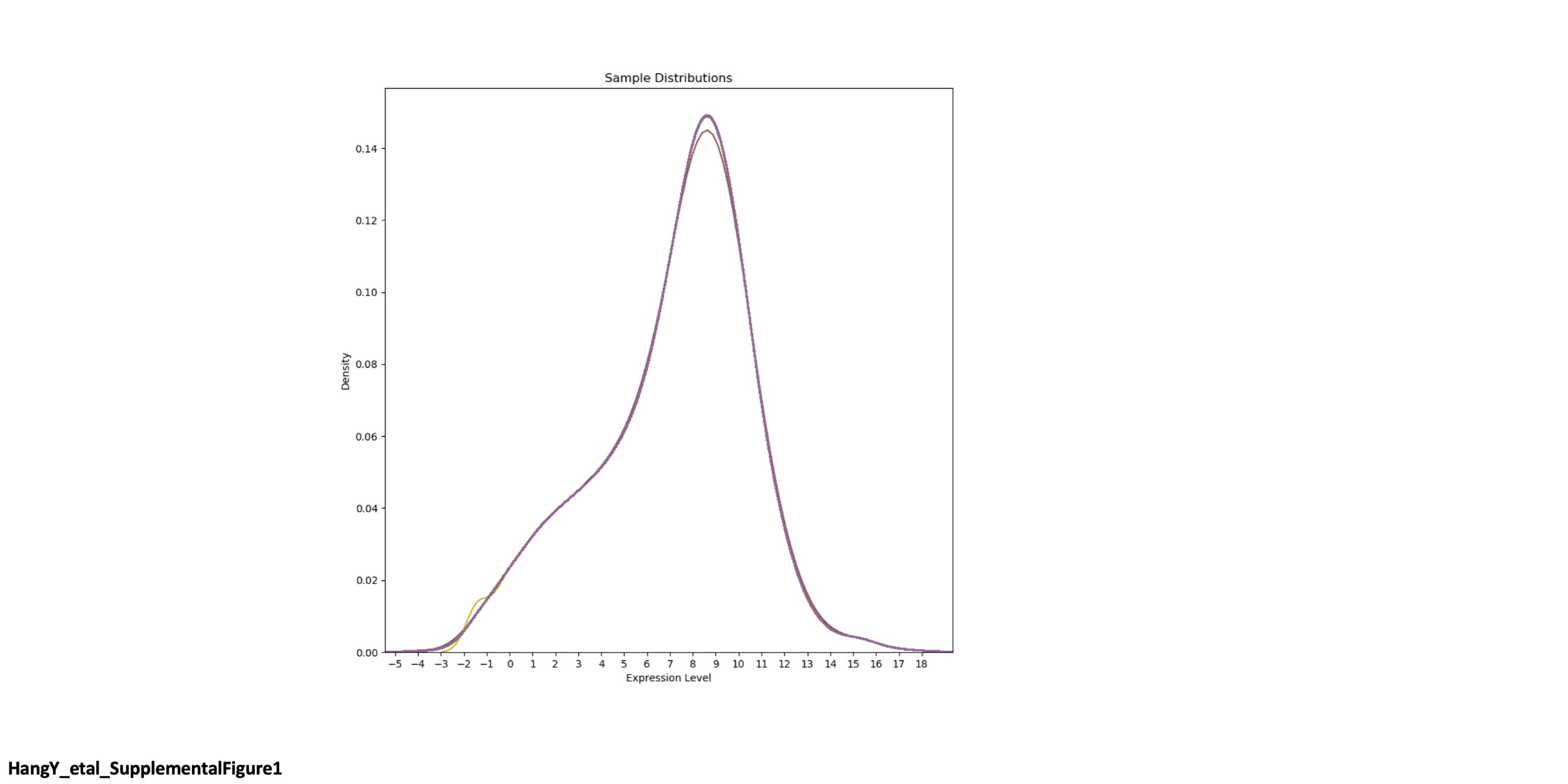
**

**Supplemental Figure 1.** Unified Lung Matrix Sample Distribution Plot. The combined lung GEM was log base 2 transformed and quantile normalized. Different color lines represent samples that have slightly different distribution. X-axis represents log base 2 transformed gene expression values, and y-axis represents the corresponding density.

**Supplemental Figure 2.** Unified Lung Sample Stage Distribution. Dark blue indicates not reported samples; Orange indicates lung cancer Stage I condition; Gray indicates lung cancer Stage II condition; Yellow indicates lung cancer Stage III condition; light blue indicates lung cancer Stage IV condition; and green indicates GTEx normal samples.


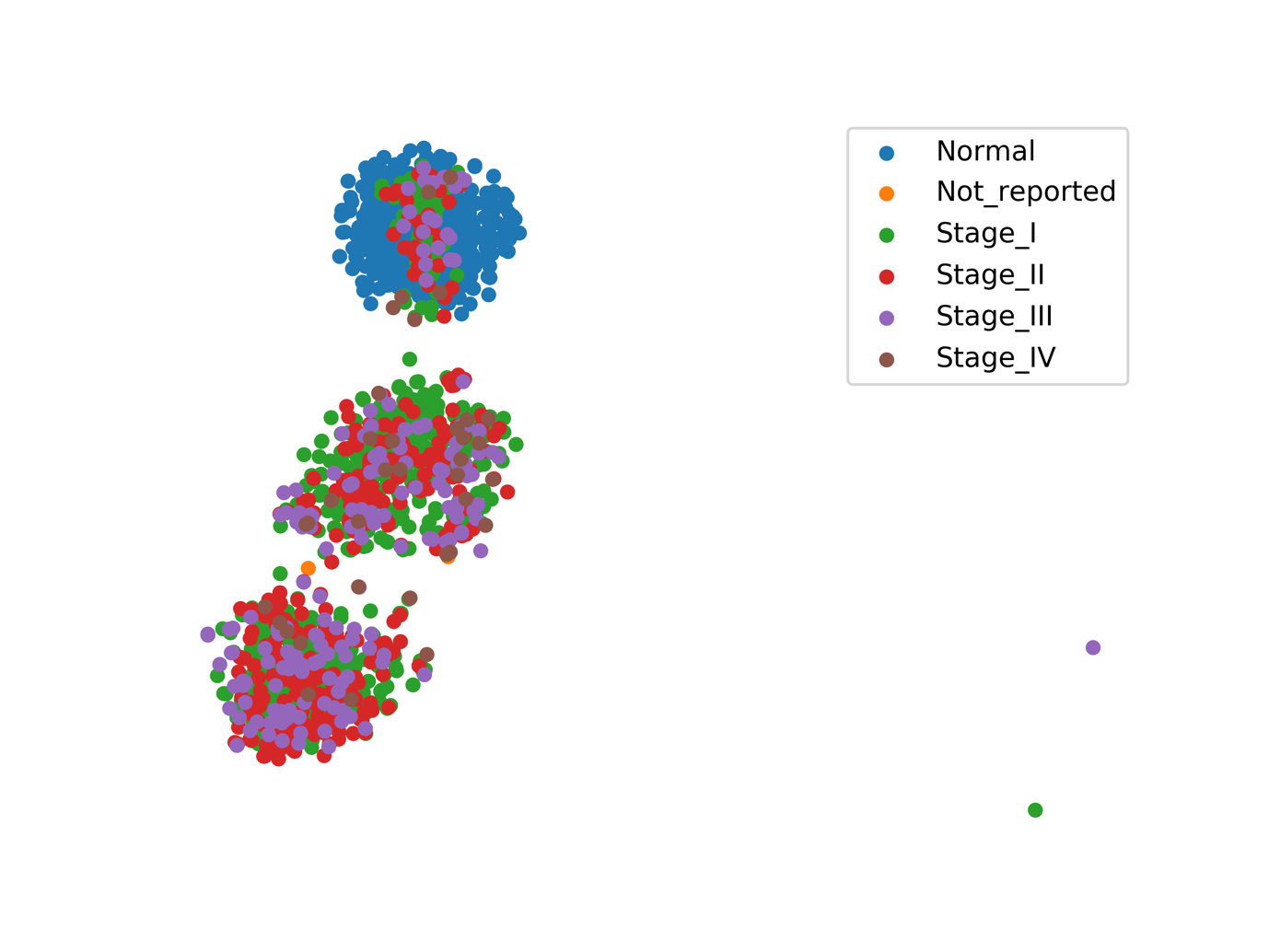
**Supplemental Figure 3.** t-SNE Visualization of Gene Expression Patterns for Unified Lung Samples Stage Information. This unified lung data contains GTEx normal, TCGA solid normal, LUAD, and LUSC samples. Stage information was added to those samples. Each color represents a different stage. GTEx normal samples are labeled as blue; Not reported samples are labeled as orange; TCGA tumor stage I, II, III, and IV samples are labeled as green, red, purple and brown respectively.


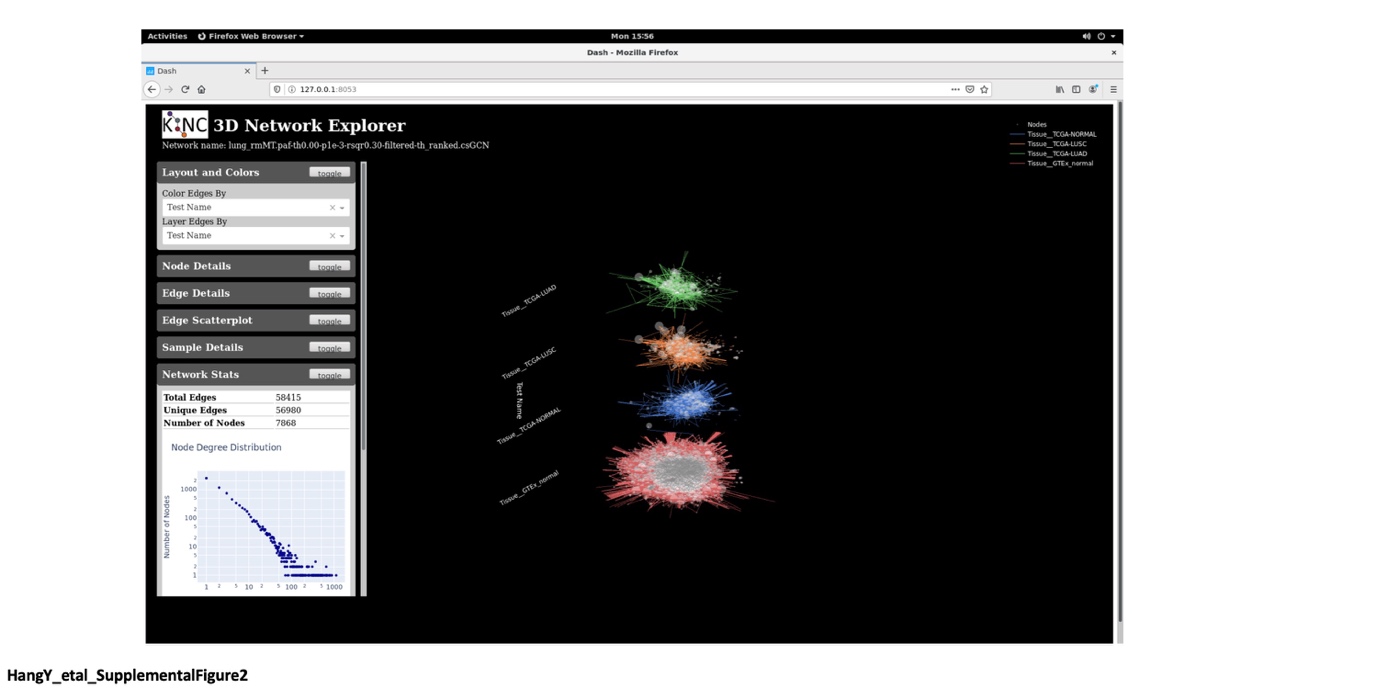
**Supplemental Figure 4.** Lung GCN 3D Network Visualization. Red represents GTEx normal specific edges; Blue represents TCGA normal specific edges; Green represents TCGA LUAD specific edges; and orange represents TCGA LUSC specific edges. The grey circle represents connectivity. Larger circles represent higher connectivity. The left part is the basic information of this network, including number of total edges, number of unique edges, number of nodes, and node degree distribution.
